# Evolution of Conformity in Social Dilemmas

**DOI:** 10.1371/journal.pone.0137435

**Published:** 2015-09-01

**Authors:** Yali Dong, Cong Li, Yi Tao, Boyu Zhang

**Affiliations:** 1 School of Statistics, Beijing Normal University, Beijing, China; 2 Département de Mathmatiques et de Statistique, Université de Montréal, Montreal, Canada; 3 Key Lab of Animal Ecology, Institute of Zoology, Chinese Academy of Sciences, Beijing, China; 4 Laboratory of Mathematics and Complex Systems, Ministry of Education, School of Mathematical Sciences, Beijing Normal University, Beijing, China; Université de Lausanne, SWITZERLAND

## Abstract

People often deviate from their individual Nash equilibrium strategy in game experiments based on the prisoner’s dilemma (PD) game and the public goods game (PGG), whereas conditional cooperation, or conformity, is supported by the data from these experiments. In a complicated environment with no obvious “dominant” strategy, conformists who choose the average strategy of the other players in their group could be able to avoid risk by guaranteeing their income will be close to the group average. In this paper, we study the repeated PD game and the repeated *m*-person PGG, where individuals’ strategies are restricted to the set of conforming strategies. We define a conforming strategy by two parameters, initial action in the game and the influence of the other players’ choices in the previous round. We are particularly interested in the tit-for-tat (TFT) strategy, which is the well-known conforming strategy in theoretical and empirical studies. In both the PD game and the PGG, TFT can prevent the invasion of non-cooperative strategy if the expected number of rounds exceeds a critical value. The stability analysis of adaptive dynamics shows that conformity in general promotes the evolution of cooperation, and that a regime of cooperation can be established in an AllD population through TFT-like strategies. These results provide insight into the emergence of cooperation in social dilemma games.

## Introduction

Classical game theory relies on the assumption of perfect rationality (i.e., players always act in a way to maximize their payoff), but in practice, people often deviate from their individual Nash equilibrium (NE). The prisoner’s dilemma (PD) game and the public goods game (PGG) are two of the well-known games in experimental economics, which show that people in the real system do not always behave rationally. Theoretically, in the PD game, mutual cooperation yields a better outcome than mutual defection but mutual defection is the only NE. The empirical studies have shown, however, that about 40% of individuals will display cooperation in the one-shot PD game. In particular, the cooperation level generally decline over time as individuals play the repeated PD game, but sometimes stable mutual cooperation can be also established [1,2]. The similar outcomes are also observed in PGG experiments. In the repeated PGG, most individuals contribute approximately half of their endowment to the common pool at the start of the game, and this average contribution will decrease to the level of approximately 20% after the game is repeated over and over [2–5].

Furthermore, the conforming behaviors widely observed in economic and psychological experiments also challenge the assumption of perfect rationality [6–9]. Unlike the rational players whose behaviors are driven by maximizing the payoff, conformists choose the average strategy of the other players in their group. Why do people match their behaviors to the group norm? One well known economic explanation is that adopting the average strategy can minimize or dilute risk because it ensures that the individual’s payoff will not be much lower than the group average [10–12]. Similarly, psychologists have also suggested that conforming behavior occurs because people desire to be liked or accepted by other group members (called normative influence), or because they desire to be correct when they are unsure of how to act (called informational influence) [8].

Conforming behavior has been applied to analyze strategies in the repeated PD game and PGG. In the repeated PD game, tit-for-tat (TFT) should be considered to be one of the most famous conforming strategies, where a player using this strategy cooperates in the first round and then does whatever the opponent did in the previous round. TFT was first introduced by Rapoport, and it is both the simplest and the most successful strategy in Axelrod's computer tournaments [13,14]. Subsequent empirical studies have found that the success of TFT is not limited to human society but that it also extends to animal populations [15]. This has encouraged biologists to explain the evolution of cooperation by reciprocal interactions based on repeated encounters [16–19]. Recently, a strategy called “moody conditional cooperation” was observed in experiments based on the spatial PD game [20–24]. The definition of this strategy comprises two main ingredients. The first is conformity, i.e. people cooperate more when more of their neighbors cooperated in the previous round. The second is that the probability that they display conforming depends on whether they cooperated or defected in the previous round. On the other hand, in repeated PGG experiments, a conformist (or a conditional cooperator) changes his/her contribution in the next round in the direction of the group average contribution of the current round [4,12,25–27]. Experimental studies have revealed that about half of the individuals in the repeated PGG can be classified as conformists [4,26,28]. Recent experiments based on PGG with institutionalized incentives shows that this proportion seems to be independent of the incentive modes. The proportion of individuals displaying conforming behavior is stabilized at around 50% in all nine treatments [12].

However, the prevalence of conforming behavior in social dilemma games raises some interesting questions. For example, why conforming behavior is so common in these games. This question has been studied in the context of an infinitely repeated PD game by analyzing adaptive dynamics in the set of reactive strategies and stochastic strategies [29–36]. The main result shows that TFT-like strategies are essential for the emergence of cooperation in a non-cooperative population, but natural selection favors generous tit-for-tat (GTFT) and win-stay lose-shift (WSLS) in the long run [30,31]. Conversely, some researchers have examined models of continuous PD game based on the linear reactive strategy method, and found that cooperative strategies such as TFT and GTFT are more difficult to invade a non-cooperative equilibrium than in the discrete PD game [37–39]. TFT and WSLS have also been generalized to discrete PGG [40–43]. In an *m*-person PGG, a TFT_k_ (0 ≤ *k* ≤ *m*) strategist cooperates if at least *k* individuals cooperated in the previous round [40–42], and a WSLS strategist cooperates if all the *m* group members cooperated or defected in the previous round [43]. Recent studies indicated that both TFT_m-1_ and WSLS can sustain cooperation in sizable group, and sometimes large group size can facilitate the evolution of cooperation [42,43].

In this paper, we study the evolution of conformity in the repeated PD game and the repeated *m*-person PGG by a selection-mutation process. In our model, a conforming strategy is defined as a 2D vector (*x*, *p*) ∈ [0, 1]^2^, where *x* describes the initial action and *p* measures the influence of the other players’ choices in the previous round. To be specific, in a repeated PD game, *x* is the probability of cooperating in the first round and *p* is the probability of imitating the opponent’s action used in the previous round (thus, with probability 1 − *p*, the player does not change his/her choice). Similarly, in the repeated PGG, *x* is the contribution level in the first round and *p* is the influence of other group members’ contributions, where a player with *p* = 0 will not change his/her contribution via rounds, and a player with *p* = 1 will always contribute the average contribution of the other members in the previous round. Following this definition, TFT and suspicious tit-for-tat (STFT) for the repeated PD game are written as (1,1) and (0,1), respectively, and AllC (i.e., always cooperate in the PD game or always full contribution in the PGG) and AllD (i.e., always defect in the PD game or always no contribution in the PGG) are (1,0) and (0,0), respectively. Depending on the payoff obtained in the repeated game, a strategy (*x*, *p*) may be adopted by more players because of natural selection (or social learning), and mutation occurs rarely in the evolutionary process. We study this process by considering the adaptive dynamics on the (*x*, *p*)-plane. In both the PD game and the PGG, we show that conditional altruistic strategies (i.e., *x* = 1 with large *p*) and unconditional selfish strategies (i.e., *x* = 0 with small *p*) are bistable if the expected number of rounds is large, where a population with high *p* moves to the cooperative boundary *x* = 1 and a population with low *p* moves to the defective boundary *x* = 0.

## Results

### The Prisoner’s Dilemma game

In the standard one-shot PD game, two players are offered a certain payoff, *R*, for mutual cooperation, and a lower payoff, *P*, for mutual defection. If one player cooperates while the other defects, then the cooperator gets the lowest payoff, *S*, and the defector gains the highest payoff, *T*. Thus, the payoffs satisfy *T* > *R* > *P* > *S*. We further make the common assumption 2*R* > *S* + *T* > 2*P*, such that mutual cooperation is the best outcome and mutual defection is the worst outcome.

Before studying the evolution of conformity in a population, we first consider a repeated PD game between two players using strategies *S*_1_ = (*x*_1_, *p*_1_) and *S*_2_ = (*x*_2_, *p*_2_) (called player 1 and player 2, respectively), where after each round there is a probability *ω* (0 ≤ *ω* < 1) that another round will be played [17–19]. Thus, the expected number of rounds is $\bar{n}=1/\left(1-\omega \right)$. The expected payoffs for two players, denoted by *E*(*S*_1_) and *E*(*S*_2_), are calculated in Section A in S1 Text. When *p*_1_ + *p*_2_ > 0, i.e., if at least one of the two players tends to conform, they will finally obtain a similar single-round expected payoff as $\bar{n}\to \infty$. This implies that conformity can help to avoid inequality between the two players [44–45]. In particular, when one of *p*_1_ and *p*_2_ equals to 1, |*E*(*S*_1_)−*E*(*S*_2_)| must be less than *T* − *S*, which is the maximal payoff difference between outcomes in the one-shot PD game [10,11]. However, when *p*_1_ + *p*_2_ = 0, the payoff difference between the two players is linearly increasing in $\bar{n}$, where the player with higher initial cooperative level is exploited by the other.

**Fig 1:**
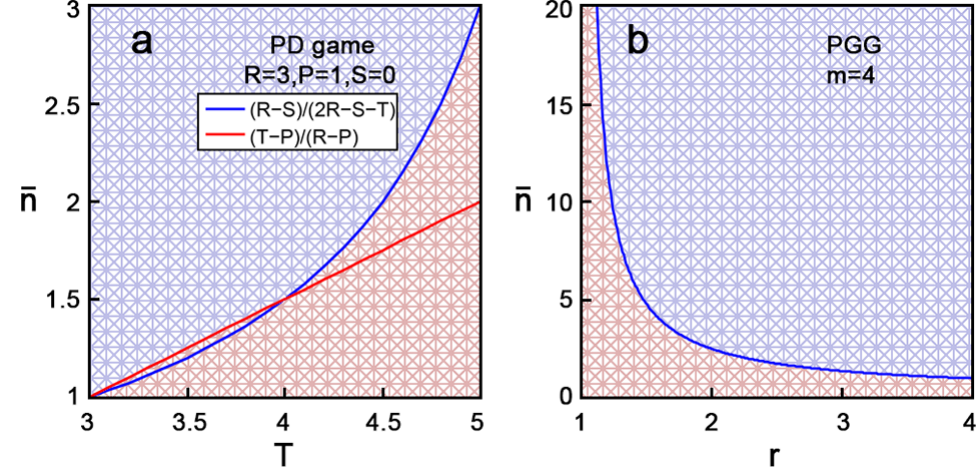
Evolutionary stabilities for TFT in the repeated PD game and the repeated PGG. **(a)** PD game with payoffs *R* = 3, *P* = 1, *S* = 0 and 3 < *T* < 5. A TFT population can prevent the invasion of any non-cooperative strategy if $\bar{n}>\text{max}\{\left(T-P\right)/\left(R-P\right),\left(R-S\right)/\left(2R-S-T\right)\}$ (the blue region). **(b)** 4-person PGG with 1 < *r* < 4. A TFT population can prevent the invasion of any non-cooperative strategy if $\bar{n}$ is above the blue curve (the blue region).

We now turn to the evolutionary stability of TFT in the set of conforming strategies. Consider a large population that consists of two types of players, *S*_1_ = (*x*_1_, *p*_1_) and *S*_2_ = (1,1) (still denoted as player 1 and player 2 for simplicity), where a small number of mutants use (*x*_1_, *p*_1_) and residents use TFT. Note that a TFT population can be invaded by AllC and other cooperative strategies such as GTFT through neutral drift, TFT is not an evolutionarily stable strategy [46–47]. We show in Section A in S1 Text, however, that a TFT population can prevent the invasion of any non-cooperative strategy (i.e., (*x*_1_, *p*_1_) with *x*_1_ < 1) if $\bar{n}>\text{max}\{\left(T-P\right)/\left(R-P\right),\left(R-S\right)/\left(2R-S-T\right)\}$ (see Fig 1A). As noted by Nowak and Sigmund, TFT plays an essential role in the emergence and maintenance of cooperation, and it paves the way for more generous strategies [19,30].

**Fig 2:**
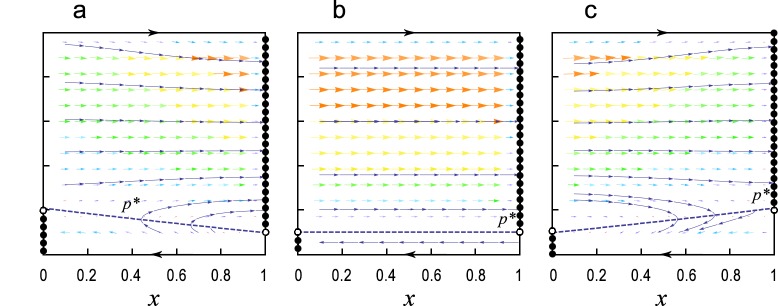
Phase portrait of the adaptive dynamics Eqs (1) and (2). For each of the three graphs, there is a curve *p* = *p* ^*^(*x*) (the blue dash curve) separating the (*x*, *p*)-plane such that *dx*/*dt* > 0 for *p* > *p* ^*^(*x*) and *dx*/*dt* < 0 for *p* < *p* ^*^(*x*). Stable equilibria and unstable equilibria of the adaptive dynamics are marked by solid dots and empty dots, respectively. Trajectories with large initial *p* converge to *x* = 1, and with small initial *p* converge to *x* = 0. **(a)** Repeated PD game with $\bar{n}=6$, *R* = 4, *P* = 2, *S* = 0 and *T* = 5. **(b)** Repeated PD game with $\bar{n}=6$, *R* = 3, *P* = 1, *S* = 0 and *T* = 4. Because *R* + *P* = *S* + *T*, there exists a critical *p* ^*^ = 0.1, where a trajectory of Eq (2) starting from (*x*, *p*) converges to (1, *p*) if *p* > *p* ^*^, and converges to (0, *p*) if *p* < *p* ^*^. **(c)** Repeated PD game with $\bar{n}=6$, *R* = 3, *P* = 1, *S* = 0 and *T* = 5.

Let us now consider the situation with mutation and investigate the evolutionary dynamics on the (*x*, *p*)-plane. Based on the standard adaptive dynamics model [29,48], we assume that mutations occur rarely and locally, where a mutant adopts a new strategy that adds a small random value on the resident strategy. This assumption implies that a mutant will either vanish or take over the population before the next mutation occurs, and the mutational jumps are small that the resident strategy changes continuously [48]. Thus, the evolution of resident strategy (*x*, *p*) in (0,1)^2^ can be described by the following adaptive dynamics:

$$\begin{array}{l} \frac{dx}{dt}=\frac{R-P}{2(1-\omega )}-\frac{T-S}{2(1-\omega (1-2p))}+(1-2x)\frac{S+T-R-P}{2(1-\omega (p^{2}+{\left(1-p\right)}^{2}))}\;,\\ \frac{dp}{dt}=(S+T-R-P)x(1-x)\frac{\omega (2p-1)}{{\left(1-\omega (p^{2}+{\left(1-p\right)}^{2})\right)}^{2}}\;, \end{array}$$ (1)

where *dx*/*dt* < 0 for *p* → 0 (see Section A in S1 Text). If *dx*/*dt* > 0 for *p* → 1 (this happens when $\bar{n}$ is large), then there exists a curve *p* = *p* ^*^(*x*) separating the (*x*, *p*)-plane such that *dx*/*dt* > 0 for *p* > *p* ^*^(*x*) and *dx*/*dt* < 0 for *p* < *p* ^*^(*x*), i.e., *x* tends to increase when *p* is large and tends to decrease when *p* is small (see Fig 2 and Section A in S1 Text). The intuition is simple: If your opponent is a conformist, then cooperating in the first round will obtain a higher payoff because your opponent will follow your choice. If the opponent is not affected by your behaviors, however, defection is the best choice. In particular, when *R* + *P* = *S* + *T*, *dx*/*dt* is independent of *x*, and *p* keeps to a constant. In this case, Eq (1) can be simplified as:

$$\begin{array}{l} \frac{dx}{dt}=\frac{R-P}{2(1-\omega )}-\frac{T-S}{2(1-\omega (1-2p))}\;,\\ \frac{dp}{dt}=0. \end{array}$$ (2)

Note that the adaptive dynamics Eqs (1) and (2) are not well defined at the boundaries *x* = 0 and *x* = 1 because *x* cannot increase (or decrease) at *x* = 1 (or *x* = 0) even if *dx*/*dt* > 0 (or *dx*/*dt* < 0). Therefore, we add two boundary conditions (i) *dx*/*dt*|_*x*=0_ = 0 if *dx*/*dt*|_*x*=0_ < 0 and (ii) *dx*/*dt*|_*x*=1_ = 0 if *dx*/*dt*|_*x*=1_ > 0 to Eqs (1) and (2) [29,48]. The dynamic properties of Eqs (1) and (2) (with the two boundary conditions) are analyzed in Section A in S1 Text (see Fig 2 for the phase portraits). The main results show that the dynamics always have a continuum of (neutral) stable defective equilibria, {(0, *p*) | 0 ≤ *p* < *p* ^*^(0)}, and a continuum of (neutral) stable cooperative equilibria, {(1, *p*) | *p* ^*^(1) < *p* ≤ 1}, exists if $\bar{n}$ is large enough such that a TFT population cannot be invaded by any non-cooperative strategy. In addition, the dynamics have two unstable equilibria, (0, *p* ^*^(0)) and (1, *p* ^*^(1)).

**Fig 3:**
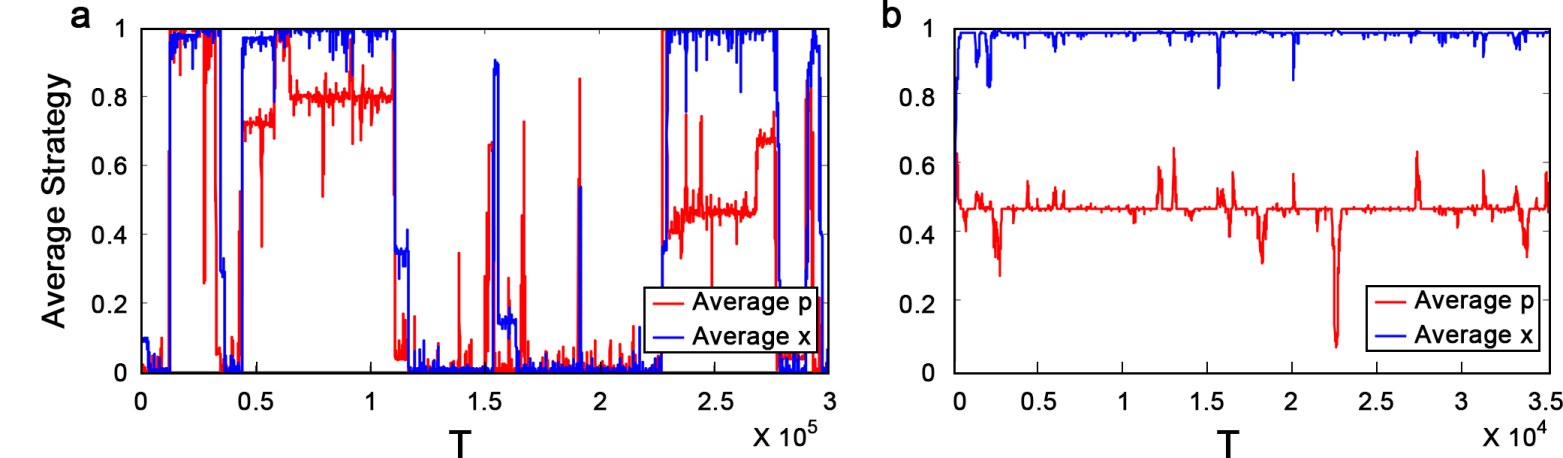
Monte-Carlo simulations for the evolution of conformity in the repeated PD game. The graphs show two typical simulation runs for a population of size 100. At the beginning of each time step, individuals are randomly divided into 50 pairs and play the repeated PD game. In each time step an average of 10 individuals are chosen to update, where they imitate actions that perform better with a probability proportional to the payoffs obtained in the repeated game (i.e., this updating process can be approximately described by the replicator dynamics [48]). In addition, with probability 0.1, one of the 100 individuals is chosen to adopt a new strategy (i.e., the average individual mutation rate is 0.001) by adding a small random value (draw from Gaussian noise (0, 0.1)) on its former strategy. **(a)** Repeated PD game with $\bar{n}=6$, *R* = 3, *P* = 1, *S* = 0 and *T* = 5. The population oscillates between the cooperative boundary *x* = 1 and the defective boundary *x* = 0. **(b)** Repeated PD game with $\bar{n}=6$, *R* = 4, *P* = 2, *S* = 0 and *T* = 5. The population can be stabilized at the cooperative boundary.

Since the adaptive dynamics Eqs (1) and (2) cannot be used to describe the change of *x* at the boundaries, we apply Monte-Carlo method to investigate the long-run evolution of (*x*, *p*) in [0, 1]^2^. If *R* + *P* ≤ *S* + *T* and $\bar{n}$ is large, the population oscillates between the boundaries *x* = 1 and *x* = 0 (see Fig 3A). To be specific, a trajectory starting from large initial *p* will first converge to the cooperative boundary. As it reaches the cooperative boundary, *p* may decrease due to neutral drift, and when *p* becomes smaller than *p* ^*^(1), the trajectory will move toward the defective boundary. In contrast, if *R* + *P* > *S* + *T*, the population can be stabilized at the cooperative boundary, because a trajectory starting from (1, *p*) with *p* slightly smaller than *p* ^*^(1) will converge to a stable cooperative equilibrium (see Figs 2C and 3B).

### Public Goods Game

In a single-round *m*-person PGG, each player in a group of size *m* is given a fixed endowment and chooses how much of that endowment to put into a common pool. The total amount in the pool is multiplied by a factor *r* with 1 < *r* < *m* and then redistributed evenly to each player in the group. It is to the group’s advantage if all players contribute their total endowment because *r* > 1, but each player, given the contributions of the others, does best by contributing nothing because *r* < *m*.

As in the repeated PD game, we consider a repeated *m*-person PGG, where after each round there is a probability *ω* (0 ≤ *ω* < 1) that another round will be played, i.e., the expected number of rounds of the PGG is $\bar{n}=1/\left(1-\omega \right)$. If a player using strategy (*x*, *p*) contributes *y*(*t*) of his/her total endowment in round *t*, and the average contribution of the other *m* − 1 players is *y* ^*^(*t*), then his/her contribution in round *t* + 1 will be *y*(*t* + 1) = *y*(*t*) + *p*(*y* ^*^(*t*) − *y*(*t*)). Let us assume that a repeated PGG consists of two types of players *S*_1_ = (*x*_1_, *p*_1_) and *S*_2_ = (*x*_2_, *p*_2_), where one player (i.e. the mutant) uses *S*_1_ and the other *m* − 1 players (i.e. the residents) use *S*_2_. We denote the expected payoffs for two types of players by *E*(*S*_1_) and *E*(*S*_2_), respectively, and calculate them in Section B in S1 Text. For conveniences, we still call (1,1) the TFT strategy in this section. It is clear that a TFT population can be invaded by cooperative strategies through neutral drift, and we show in Section B in S1 Text that a TFT population can prevent the invasion of any non-cooperative strategies if and only if $\bar{n}>\left(m^{2}-2m+r\right)/(r-1)m$ (see Fig 1B). In particular, when *m* = 2, the repeated PGG is equivalent to a continuous PD game with payoffs (*R*, *S*, *T*, *P*) = (*r*, *r*/2, *r*/2+1, 1) [37,38], and TFT can maintain cooperation if and only if $\bar{n}>r/2(r-1)$. This condition is consistent with that of the discrete PD game.

**Fig 4:**
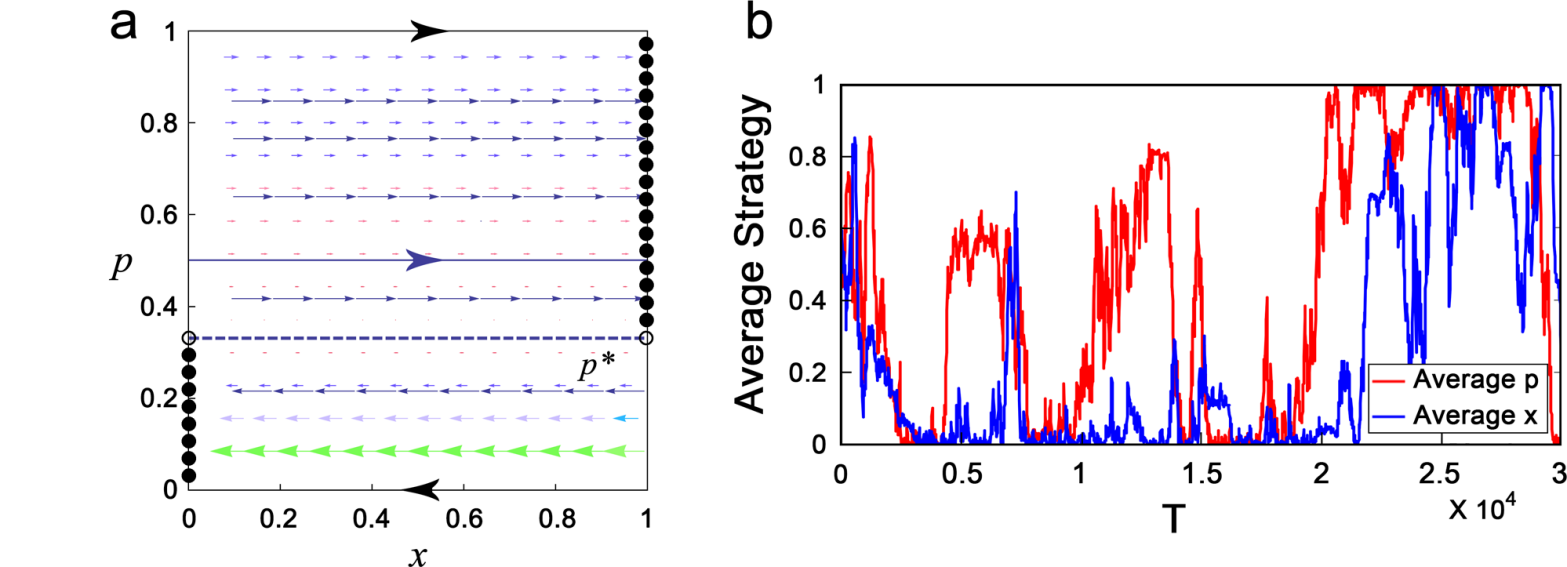
The evolution of conformity in the repeated PGG. Repeated PGG with *n* = 10, *m* = 4 and *r* = 1.6. **(a)** Phase portrait of the adaptive dynamics Eq (4). Stable equilibria and unstable equilibria are marked by solid dots and empty dots, respectively.*p* ^*^ = 1/3 (the blue dash line). A trajectory of Eq (4) starting from (*x*, *p*) converges to the stable cooperative equilibrium (1, *p*) if *p* > *p* ^*^, and converges to the unstable defective equilibrium (0, *p*) if *p* < *p* ^*^. **(b)** Monte-Carlo simulation result for a population of size 100. At the beginning of each time step, individuals are randomly divided into 25 groups and play the repeated PGG. In each time step, an average of 10 individuals are chosen to update, where they imitate actions that perform better with a probability proportional to the payoffs obtained in the repeated PGG. With probability 0.1, one of the 100 individuals is chosen to adopt a new strategy (i.e., the average individual mutation rate is 0.001) by adding a small random value (draw from Gaussian noise (0, 0.1)) on its former strategy. Monte-Carlo simulation also shows that conditional altruistic strategies (i.e., *x* = 1 with large *p*) and unconditional selfish strategies (i.e., *x* = 1 with small *p*) are bistable, and the population oscillates between *x* = 1 and *x* = 0.

We now consider a large homogeneous population with (resident) strategy (*x*, *p*), and we assume that the population moves towards the direction in which mutants have the higher invasion payoff. Then, the resulting adaptive dynamics on the (*x*, *p*)-plane is given by

$$\begin{array}{l} \frac{dx}{dt}=\frac{\left(r-1\right)}{\left(1-\omega \right)m}-\frac{\left(m-1\right)}{m\left(1-\omega \left(1-\frac{pm}{m-1}\right)\right)}\;,\\ \frac{dp}{dt}=0, \end{array}$$ (3)

with two boundary conditions (i) *dx*/*dt*|_*x*=0_ = 0 if *dx*/*dt*|_*x*=0_ < 0 and (ii) *dx*/*dt*|_*x*=1_ = 0 if *dx*/*dt*|_*x*=1_ > 0. Remarkably, the first equation of Eq (3) is independent of *x*, and the second equation states that *p* keeps to be a constant. Thus, similarly to the dynamic behavior of Eq (2), there exists a critical *p* ^*^, where a trajectory of Eq (3) starting from (*x*, *p*) converges to the stable cooperative equilibrium (1, *p*) if *p* > *p* ^*^, and converges to the stable defective equilibrium (0, *p*) if *p* < *p* ^*^ (see Fig 4A and Section B in S1 Text). In addition to adaptive dynamics, Monte-Carlo simulation also shows that conditional altruistic strategies (i.e., *x* = 1 with large *p*) and unconditional selfish strategies (i.e., *x* = 1 with small *p*) are bistable, and the population oscillates between *x* = 1 and *x* = 0 (see Fig 4B).

## Discussion

Conditional cooperation or conformity has been widely observed in repeated social dilemma experiments [4,12,20–28]. The prevalence of conformity in these experiments agrees with the fact that this behavior is common in nature and human society [49,50]. In a complicated environment with no obvious “dominant” strategy, conformists should be able to avoid or dilute risk by guaranteeing that their income will be close to the group average. In this paper, we study the repeated PD game and the repeated *m*-person PGG by restricting individual strategies to the set of conforming strategies. We are particularly interested in TFT strategy. In both the PD game and the PGG, TFT can prevent the invasion of non-cooperative strategies if the expected number of rounds, $\bar{n}$, exceeds a critical value. We then investigate the adaptive dynamics in the (*x*, *p*)-plane (i.e. the set of conforming strategies). Stability analysis shows that conformity in general promotes the evolution of cooperation. Trajectories of the adaptive dynamics starting from initial values with large *p* converge to the cooperative boundary *x* = 1. In particular, if the payoffs of the PD game satisfy *R* + *P* > *S* + *T* and $\bar{n}$ is large enough, cooperation can be stabilized under the influence of selection and mutation. In contrast, in the PD game with *R* + *P* ≤ *S* + *T* and the PGG, the population oscillates between the cooperative state and the defective state.

In our paper, a conforming strategy is defined by a 2D vector (*x*, *p*). In the repeated PD game, an important class of strategies consists of so-called “memory-one” strategies (or stochastic strategies) [29]. A memory-one strategy is written as a 5D vector (*x*, *p*_*CC*_, *p*_*CD*_, *p*_*DC*_, *p*_*DD*_), where *x* is the probability of cooperating in the first round and *p*_*CC*_, *p*_*CD*_, *p*_*DC*_ and *p*_*DD*_ are the conditional probabilities of playing C after CC, CD, DC and DD interactions, respectively. Within the 5D unit cube of all memory-one strategies, the conforming strategies form a 2D subset containing strategies such as AllC, AllD, TFT and STFT (but WSLS and GTFT are not included). Evolutionary simulations based on the set of all memory-one strategies showed that TFT-like mutants can invade AllD population, after which TFT will be replaced by more generous strategies such as GTFT and AllC; finally, the population will be undermined by WSLS [31]. The last two steps in the above process are easy to be understood: TFT is replaced by GTFT and AllC because TFT cannot correct mistakes in a noisy environment, and AllC is dominated by WSLS because WSLS can exploit cooperators. However, the first step is counter intuitive because AllD and TFT are bistable under deterministic evolutionary dynamics [18,19,48]. A great deal of research has been devoted to explaining how TFT can establish a regime of cooperation in an AllD population [30,42,51–53]. Our paper notes an evolutionary path from AllD to TFT-like strategies. As shown in Figs 2 and 4, selfish conformists (e.g. STFT-like strategies) can invade an AllD population through neutral drift. When most of the players become selfish conformists, cooperative conformists (e.g. TFT-like strategies) will obtain a higher payoff. The population will then evolve to a conditional cooperative regime, which cannot be invaded by non-cooperative strategies.

On the other hand, in the continuous PGG, the conforming strategies are included in the set of “conditional contribution” strategies introduced by Fischbacher and Gächter [4]. To be specific, a conditional contribution strategy is described by a 4D vector (*x*, *a*, *b*, *c*), where *x* is the contribution rate in the first round and the contribution rate in round *t* + 1 is given by *y*(*t* + 1) = *ay* ^*^(*t*) + *by*(*t*) + *c*, where *y* ^*^(*t*) is the average contribution of the other group members in round *t*. Following this model, a conforming strategy (*x*, *p*) can be represented by (*x*, *p*, (1 − *p*), 0). Furthermore, in a two-person discrete PGG, strategy (1,1) behaves same as the TFT strategy of the repeated PD game (that is why we still call (1,1) TFT). However, when *m* > 2, TFT_k_ strategies of the discrete PGG cannot be expressed by conforming strategies [40–42].

It is worthwhile to note that our theoretical predictions are consistent with the observations in recent network PD experiments and repeated PGG experiments. In the network PD experiments, payoffs are taken as (*R*,*S*,*T*,*P*) = (7,0,10,0) (i.e., *R* + *P* < *S* + *T*) and (*R*,*S*,*T*,*P*) = (3,0,4,1) (i.e., *R* + *P* = *S* + *T*) [20–22]. In these experiments, Grujić et al. observed two typical strategies, conditional cooperation and unconditional defection, and the population evolved to full defection at the end of the games [23–24]. Our simulation result verifies that these two strategies cannot coexist, and the population will converge to either the cooperative boundary or the defective boundary (see Figs 2B, 2C and 3B), although our model cannot be applied to analyze their experiments directly because network structures may play an essential role in decision making process. On the other hand, Fischbacher and Gächter noted that people’s behaviors in repeated PGG experiments can be explained by a combination of their own beliefs and the observation of others’ contributions [4]. They then developed the “conditional contribution” model, and used a 4D vector (*x*, *a*, *b*, *c*) to characterize individual strategy. Note that in their experiments, the sum of *a* and *b* is insignificantly different from 1 (*a* = 0.415, *b* = 0.569 and *a* + *b* = 0.984) and *c* is small (*c* = 0.118). Thus, people’s behaviors in their experiments can be approximately described by our model with *p* = 0.415. In particular, they observed that individual strategies in different groups have a large degree of heterogeneity, where unconditional free-riding and conditional cooperation are the two largest types. This exactly matches our theoretical result that conditional altruistic strategies and unconditional selfish strategies are both stable in the repeated PGG.

We analyze the impact of conformity on the evolution of cooperation in social dilemmas by considering that players’ choices are affected by the options of their group members. In more realistic populations, different individuals interact with different subsets of the entire population, a type of structure that can be described by means of complex networks. It is well known that network reciprocity can promote cooperation in evolutionary social dilemmas [54–58]. Recent studies indicated that conformity enhances network reciprocity on rings and square lattices because conformists can form an effective surface tension around cooperative clusters that prevent the invasion of defectors [59,60]. However, conformity hinders the evolution of cooperation on scale-free networks [59]. Another study based on a network coordination game showed that the existence of conformists prevents the freezing of the network in domains with different conventions, thus leading to global consensus [61]. Furthermore, an appropriate fraction of conformists can release the zero-sum competition in a network extension of matching pennies, and the population will evolve to pure Nash equilibria [62,63]. In the above studies, conformists are assumed to adopt the most common strategy in their neighborhoods, but the evolutionary origin of this behavioral rule has not been explained. Therefore, an interesting question in the future is to consider the evolution of conformity in social networks. This may help us to understand individual behaviors in networks based PD and PGG experiments and why a static network structure has a limited effect on sustaining cooperation [20–22,64–67].

Another possible development would be to study the evolutionary competition between different behavioral rules [60]. To be specific, in the evolutionary game, players can change not only their actions but also the motivations behind their actions. The behavioral rules we mentioned here include not only well-known best response, payoff-driven imitation (e.g. imitate-the-best and imitate-if-better), aspiration-driven updating or conformity but also some recently discovered and more delicate strategies, such as extortionate strategies that allow a player to perform above the average payoff of the group, generous strategies that let a player perform below the average, and fair strategies that ensure that their own payoff matches the average [32–36,44,45]. A recent empirical study observed that although extortionists succeeded against each of their opponents, extortionate strategies resulted in lower payoffs than generous strategies and TFT in the long-run because most of human subjects adopted TFT-like strategies [28]. As noted by Duersch et al., TFT (and some payoff-driven imitation rules) can hardly be beaten, even by very sophisticated opponents, in repeated PD games and PGG in the sense that there is no strategy that can exploit them as a money pump [10,11]. We expect that the exploration of the competition between different behavioral rules can provide insight into the prevalence of conforming behavior in nature and human society.

## Supporting Information

S1 Text Supporting Information for “Evolution of conformity in social dilemmas”. (DOC)
