# Supplementary material for: Evolution of Conformity in Social Dilemmas
Source: PLoS One. 2015 Sep 1;10(9):e0137435. doi: 10.1371/journal.pone.0137435 (PMC4556697; doi:10.1371/journal.pone.0137435)
Supplement: S1 Text — (DOC) [file pone.0137435.s001.doc]

**Supporting Information for “Evolution of conformity in social dilemmas”**

**Yali Dong^1 $^, Cong Li^2 $^, Yi Tao^3^, Boyu Zhang^4*^**

^1^School of Statistics, Beijing Normal University, Beijing, China

^2^Département de Mathmatiques et de Statistique, Université de Montréal, Montreal, Canada

^3^Key Lab of Animal Ecology, Institute of Zoology, Chinese Academy of Sciences, Beijing, China

^4^Laboratory of Mathematics and Complex Systems, Ministry of Education, School of Mathematical Sciences, Beijing Normal University, Beijing, China

^$^The two authors have contributed equally to this paper.

^*^Corresponding author: zhangby@bnu.edu.cn

**A. The repeated Prisoner’s Dilemma game**

**Payoff calculation**

We consider a repeated PD game between two players using strategies $S_{1}=(x_{1},p_{1})$ and $S_{2}=(x_{2},p_{2})$ (call them player 1 and player 2, respectively), where after each round there is a probability $\omega$ ($0\leq\omega<1$) that another round will be played. Thus, the expected number of rounds is $\bar{n}=1/(1-\omega)$. In terms of memory-one strategy, $S_{i}$ ($i=1,2$) can be represented as a 5D vector $(x_{i},1,1-p_{i},p_{i},0)$, where $x_{i}$ is probability to cooperate in the first round, and the probabilities of choosing cooperation after CC, CD, DC and DD interactions are $1$, $1-p_{i}$, $p_{i}$ and $0$, respectively. We denote the probability that player 1 choosing action X and player 2 choosing action Y in the $k$-th round by $x_{XY}(k)$ ($X,Y\in\{C,D\}$). Thus, in the first round, we have $x_{CC}(1)=x_{1}x_{2}$, $x_{CD}(1)=x_{1}(1-x_{2})$, $x_{DC}(1)=(1-x_{1})x_{2}$ and $x_{DD}(1)=(1-x_{1})(1-x_{2})$. In round $k+1$, $x_{CC}(k+1)$, $x_{CD}(k+1)$, $x_{DC}(k+1)$ and $x_{DD}(k+1)$ satisfy the following equation

$\left(\begin{matrix} x_{CC}(k+1) \\ x_{CD}(k+1) \\ x_{DC}(k+1) \\ x_{DD}(k+1) \end{matrix}\right)=\left(\begin{matrix} 1 & (1-p_{1})p_{2} & p_{1}(1-p_{2}) & 0 \\ 0 & (1-p_{1})(1-p_{2}) & p_{1}p_{2} & 0 \\ 0 & p_{1}p_{2} & (1-p_{1})(1-p_{2}) & 0 \\ 0 & p_{1}(1-p_{2}) & (1-p_{1})p_{2} & 1 \end{matrix}\right)\left(\begin{matrix} x_{CC}(k) \\ x_{CD}(k) \\ x_{DC}(k) \\ x_{DD}(k) \end{matrix}\right).$ (S1)

Notice that the changes of $x_{CD}(k)$ and $x_{DC}(k)$ are independent of $x_{CC}(k)$ and $x_{DD}(k)$, i.e.,

$\begin{matrix} x_{CD}(1)=x_{1}(1-x_{2}), \\ x_{CD}(k+1)=(1-p_{1})(1-p_{2})x_{CD}(k)+p_{1}p_{2}x_{DC}(k), \\ x_{DC}(1)=(1-x_{1})x_{2}, \\ x_{DC}(k+1)=p_{1}p_{2}x_{CD}(k)+(1-p_{1})(1-p_{2})x_{DC}(k), \end{matrix}$ (S2)

we first derive the expressions of $x_{CD}(k)$ and $x_{DC}(k)$ according to Eq.(S2). Suppose that

$x_{CD}(k+1)+\alpha x_{DC}(k+1)=\beta(x_{CD}(k)+\alpha x_{DC}(k)),$ (S3)

where $\alpha$ and $\beta$ are undetermined coefficients. From Eq.(S2), we obtain two groups of coefficients: $\alpha_{1}=1$, $\beta_{1}=p_{1}p_{2}+(1-p_{1})(1-p_{2})$, and $\alpha_{2}=-1$, $\beta_{2}=1-p_{1}-p_{2}$. Thus, $x_{CD}(k)$ and $x_{DC}(k)$ satisfy the following recursive equations

$\begin{matrix} x_{CD}(k+1)+x_{DC}(k+1)=\beta_{1}(x_{CD}(k)+x_{DC}(k)), \\ x_{CD}(k+1)-x_{DC}(k+1)=\beta_{2}(x_{CD}(k)-x_{DC}(k)), \end{matrix}$ (S4)

and the general formulas of $x_{CD}(k)$ and $x_{DC}(k)$ can be obtained

$\begin{matrix} x_{CD}(k)=\frac{\beta_{1}^{k-1}(x_{CD}(1)+x_{DC}(1))+\beta_{2}^{k-1}(x_{CD}(1)-x_{DC}(1))}{2} \\ =\frac{(x_{1}+x_{2}-2x_{1}x_{2})}{2}(p_{1}p_{2}+(1-p_{1})(1-p_{2}))^{k-1}+\frac{x_{1}-x_{2}}{2}(1-p_{1}-p_{2})^{k-1}, \\ x_{DC}(k)=\frac{\beta_{1}^{k-1}(x_{CD}(1)+x_{DC}(1))-\beta_{2}^{k-1}(x_{CD}(1)-x_{DC}(1))}{2} \\ =\frac{(x_{1}+x_{2}-2x_{1}x_{2})}{2}(p_{1}p_{2}+(1-p_{1})(1-p_{2}))^{k-1}-\frac{x_{1}-x_{2}}{2}(1-p_{1}-p_{2})^{k-1}. \end{matrix}$ (S5)

Furthermore, notice that $x_{CC}(k)$ and $x_{DD}(k)$ satisfy

$\begin{matrix} x_{CC}(k+1)=x_{CC}(k)+(1-p_{1})p_{2}x_{CD}(k)+p_{1}(1-p_{2})x_{DC}(k), \\ x_{DD}(k+1)=x_{DD}(k)+p_{1}(1-p_{2})x_{CD}(k)+(1-p_{1})p_{2}x_{DC}(k), \end{matrix}$ (S6)

the general formulas of $x_{CC}(k)$ and $x_{DD}(k)$ are

$\begin{matrix} x_{CC}(k)=x_{1}x_{2}+\frac{(x_{1}+x_{2}-2x_{1}x_{2})}{2}[1-(p_{1}p_{2}+(1-p_{1})(1-p_{2}))^{k-1}] \\ -\frac{x_{1}-x_{2}}{2}\frac{p_{1}-p_{2}}{p_{1}+p_{2}}[1-(1-p_{1}-p_{2})^{k-1}], \\ x_{DD}(k)=(1-x_{1})(1-x_{2})+\frac{(x_{1}+x_{2}-2x_{1}x_{2})}{2}[1-(p_{1}p_{2}+(1-p_{1})(1-p_{2}))^{k-1}] \\ +\frac{x_{1}-x_{2}}{2}\frac{p_{1}-p_{2}}{p_{1}+p_{2}}[1-(1-p_{1}-p_{2})^{k-1}]. \end{matrix}$ (S7)

From Eqs.(S5) and (S7), the expected frequencies of CC, CD, DC and DD interactions in the repeated PD game, denoted by $E_{CC}$, $E_{CD}$, $E_{DC}$ and $E_{DD}$, are given by $E_{CC}=\sum_{n=1}^{\infty} (1-\omega)\omega^{n-1}\sum_{k=1}^{n} x_{CC}(k)$, $E_{CD}=\sum_{n=1}^{\infty} (1-\omega)\omega^{n-1}\sum_{k=1}^{n} x_{CD}(k)$, $E_{DC}=\sum_{n=1}^{\infty} (1-\omega)\omega^{n-1}\sum_{k=1}^{n} x_{DC}(k)$ and $E_{DD}=\sum_{n=1}^{\infty} (1-\omega)\omega^{n-1}\sum_{k=1}^{n} x_{DD}(k)$, respectively.

For convenience, denote the payoff to player $i$ when it meets player $j$ by $E_{ij}$. Thus, in the repeated PD game between player 1 and player 2, expected payoff for player 1, $E(S_{1})$ (i.e., $E_{12}$), is written as

$\begin{matrix} E_{12}=RE_{CC}+SE_{CD}+TE_{DC}+PE_{DD} \\ =(S+T-R-P)\frac{x_{1}+x_{2}-2x_{1}x_{2}}{2(1-\omega(p_{1}p_{2}+(1-p_{1})(1-p_{2})))}+(S-T)\frac{x_{1}-x_{2}}{2(1-\omega(1-p_{1}-p_{2}))} \\ +(R-P)\frac{p_{1}-p_{2}}{p_{1}+p_{2}}\left[\frac{x_{1}-x_{2}}{2(1-\omega(1-p_{1}-p_{2}))}-\frac{x_{1}-x_{2}}{2(1-\omega)}\right]+R\frac{x_{1}+x_{2}}{2(1-\omega)}+P\frac{2-x_{1}-x_{2}}{2(1-\omega)} \end{matrix}$ (S8)

and expected payoff for player 2, $E(S_{2})$ (i.e., $E_{21}$), is written as

$\begin{matrix} E_{21}=RE_{CC}+TE_{CD}+SE_{DC}+PE_{DD} \\ =(S+T-R-P)\frac{x_{1}+x_{2}-2x_{1}x_{2}}{2(1-\omega(p_{1}p_{2}+(1-p_{1})(1-p_{2})))}-(S-T)\frac{x_{1}-x_{2}}{2(1-\omega(1-p_{1}-p_{2}))} \\ +(R-P)\frac{p_{1}-p_{2}}{p_{1}+p_{2}}\left[\frac{x_{1}-x_{2}}{2(1-\omega(1-p_{1}-p_{2}))}-\frac{x_{1}-x_{2}}{2(1-\omega)}\right]+R\frac{x_{1}+x_{2}}{2(1-\omega)}+P\frac{2-x_{1}-x_{2}}{2(1-\omega)}. \end{matrix}$ (S9)

Furthermore, when both players are using $S_{1}$, their payoff is

$E_{11}=\frac{Rx_{1}+P(1-x_{1})}{1-\omega}+(S+T-R-P)\frac{x_{1}(1-x_{1})}{1-\omega(p_{1}^{2}+(1-p_{1})^{2})},$ (S10)

and when both players are using $S_{2}$

$E_{22}=\frac{Rx_{2}+P(1-x_{2})}{1-\omega}+(S+T-R-P)\frac{x_{2}(1-x_{2})}{1-\omega(p_{2}^{2}+(1-p_{2})^{2})}.$ (S11)

When $p_{1}+p_{2}>0$, the difference between the single-round expected payoffs of the two players converges to $0$ as $\bar{n}\to\infty$, i.e.,

$\lim_{\bar{n}\to\infty} \left|\frac{E_{12}-E_{21}}{\bar{n}}\right|=\lim_{\omega\to1} \left|\frac{(1-\omega)(S-T)(x_{1}-x_{2})}{1-\omega(1-p_{1}-p_{2})}\right|=0.$ (S12)

In particular, when one of $p_{1}$ and $p_{2}$ equals to $1$, we have $\left|E_{12}-E_{21}\right|<\left|(S-T)(x_{1}-x_{2})\right|\leq T-S$. However, when $p_{1}+p_{2}=0$, $\left|E_{12}-E_{21}\right|=\left|\bar{n}(S-T)(x_{1}-x_{2})\right|$, i.e., the payoff difference between the two players is linearly increasing in $\bar{n}$.

**Evolutionary stability of TFT**

Consider a large population that consists of two types of players, $S_{1}=(x_{1},p_{1})$ and $S_{2}=(1,1)$ (i.e., TFT), where the frequency of player 1 is denoted by$y$. The average payoffs of player 1 and player 2 in this population are then written as $E(S_{1})=yE_{11}+(1-y)E_{12}$ and $E(S_{2})=yE_{21}+(1-y)E_{22}$, respectively. Thus, a TFT population can prevent the invasion of a non-cooperative strategy $(x_{1},p_{1})$ with $x_{1}<1$ if $E_{22}>E_{12}$. From Eqs.(S8) and (S11),

$E_{22}-E_{12}=\frac{1-x_{1}}{2}\left(\frac{R-P}{1-\omega}\frac{1+\omega}{1+\omega p_{1}}-\frac{S+T-R-P}{1-\omega p_{1}}+\frac{S-T}{1+\omega p_{1}}\right),$ (S13)

where the sign of $E_{22}-E_{12}$ is independent of $x_{1}$, but depends on $p_{1}$ only. Notice that both $1+\omega p_{1}$ and $1-\omega p_{1}$ are positive, $E_{22}-E_{12}>0$ if and only if $(1+\omega p_{1})(1-\omega p_{1})(E_{22}-E_{12})>0$, i.e.,

$\frac{1-x_{1}}{2}\left[\omega p_{1}\left(R+P-2S-(R-P)\frac{1+\omega}{1-\omega}\right)-\left(2T-R-P-(R-P)\frac{1+\omega}{1-\omega}\right)\right]>0.$ (S14)

Notice that the left hand side of inequality Eq.(14) is a linear function of $p_{1}$, $E_{22}-E_{12}>0$ for all $p_{1}\in[0,1]$ and $x_{1}\in[0,1)$ if and only if the TFT population can prevent the invasion of the two extreme strategies AllD $(0,0)$ and STFT $(0,1)$. For $S_{1}=(0,0)$, $E_{22}-E_{12}>0$ if and only if $\bar{n}>(T-P)/(R-P)$, and for $S_{1}=(0,1)$, $E_{22}-E_{12}>0$ if and only if $\bar{n}>(R-S)/(2R-S-T)$. Furthermore, $(T-P)/(R-P)>(R-S)/(2R-S-T)$ (i.e., the first condition is stronger than the second) if and only if $T+S<R+P$.

**Evolutionary dynamics**

The replicator equation for the two types of players, $S_{1}=(x_{1},p_{1})$ and $S_{2}=(x_{2},p_{2})$ is written as

$\begin{matrix} \frac{dy}{dt}=y(1-y)(E(S_{1})-E(S_{2})) \\ =y(1-y)\left[y(E_{11}-E_{21})+(1-y)(E_{21}-E_{22})\right], \end{matrix}$ (S15)

where $E(S_{1})=yE_{11}+(1-y)E_{12}$ and $E(S_{2})=yE_{21}+(1-y)E_{22}$. When $p_{1}=p_{2}=p$, we have

$\begin{matrix} E_{11}-E_{21}=\frac{x_{1}-x_{2}}{2}\left[\frac{R-P}{1-\omega}-\frac{T-S}{1-\omega(1-2p)}+\frac{(S+T-R-P)(1-2x_{1})}{1-\omega(p^{2}+(1-p)^{2})}\right], \\ E_{12}-E_{22}=\frac{x_{1}-x_{2}}{2}\left[\frac{R-P}{1-\omega}-\frac{T-S}{1-\omega(1-2p)}+\frac{(S+T-R-P)(1-2x_{2})}{1-\omega(p^{2}+(1-p)^{2})}\right], \end{matrix}$ (S16)

i.e., $E_{11}-E_{21}=E_{12}-E_{22}+O((x_{1}-x_{2})^{2})$. Thus, if $S_{1}$ and $S_{2}$ only have small difference on $x$ and no difference on $p$, $E_{11}>E_{21}$ if and only if $E_{12}>E_{22}$. This implies that if a player 1 can invade a population of player 2, then it will replace the population under the replicator equation Eq.(S15). On the other hand, when $x_{1}=x_{2}=x$, we have

$\begin{matrix} E_{11}-E_{21}=(S+T-R-P)x(1-x)\left(\frac{1}{1-\omega(p_{1}^{2}+(1-p_{1})^{2})}-\frac{1}{1-\omega(p_{1}p_{2}+(1-p_{1})(1-p_{2}))}\right) \\ E_{12}-E_{22}=(S+T-R-P)x(1-x)\left(\frac{1}{1-\omega(p_{1}p_{2}+(1-p_{1})(1-p_{2}))}-\frac{1}{1-\omega(p_{2}^{2}+(1-p_{2})^{2})}\right) \end{matrix}$ (S16)

Notice that $1/\left[1-\omega(p_{1}p_{2}+(1-p_{1})(1-p_{2}))\right]$ is between $1/\left[1-\omega(p_{1}^{2}+(1-p_{1})^{2})\right]$ and $1/\left[1-\omega(p_{2}^{2}+(1-p_{2})^{2})\right]$ for $p_{1},p_{2}<1/2$ and $p_{1},p_{2}>1/2$, $E_{11}-E_{21}$ and $E_{12}-E_{22}$ have the same sign almost always when $p_{1}\approx p_{2}$ (the only exception is $p_{1}\approx p_{2}=1/2$). Thus, if $S_{1}$ and $S_{2}$ only have small difference on $p$ and no difference on $x$, we also have $E_{11}>E_{21}$ if and only if $E_{12}>E_{22}$, i.e., a single player 1 will replace a population of player 2 if it can invade.

Let us now add the possibility of mutation and derive the adaptive dynamics on the $(x,p)$-plane. If mutation occurs on $x$, i.e., the mutant strategy $S_{1}$ satisfies $x_{1}\approx x_{2}$ and $p_{1}=p_{2}=p$, then it can replace the resident population if and only if $E_{12}-E_{22}>0$. Thus, from Eq.(S15), the adaptive dynamics of $x$ can be written as

$\frac{dx}{dt}=\frac{\partial\left(E_{12}-E_{22}\right)}{\partial x_{1}}\left|_{x_{1}=x_{2}=x}\right.=\frac{R-P}{2(1-\omega)}-\frac{T-S}{2(1-\omega(1-2p))}+(1-2x)\frac{S+T-R-P}{2(1-\omega(p^{2}+(1-p)^{2}))}.$ (S17)

Note that the adaptive dynamics Eq.(17) cannot be used to describe the change of $x$ at the boundaries $x=0$ and $x=1$ because $x$ cannot increase (or decrease) at $x=1$ (or $x=0$) even if $dx/dt>0$ (or $dx/dt<0$). Therefore, we add two boundary conditions (i) $dx/dt\left|_{x=0}\right.=0$ if $dx/dt\left|_{x=0}\right.<0$ and (ii) $dx/dt\left|_{x=1}\right.=0$ if $dx/dt\left|_{x=1}\right.>0$. On the other hand, if mutation occurs on $p$, i.e., the mutant strategy $S_{1}$ satisfies $p_{1}\approx p_{2}$ and $x_{1}=x_{2}=x$, the adaptive dynamics of $p$ can be written as

$\frac{dp}{dt}=\frac{\partial\left(E_{12}-E_{22}\right)}{\partial p_{1}}\left|_{p_{1}=p_{2}=p}\right.=(S+T-R-P)x(1-x)\frac{\omega(2p-1)}{(1-\omega(p^{2}+(1-p)^{2}))^{2}}.$ (S18)

We first analyze the dynamic behavior of Eq.(S17) (i.e., the first equation of Eq.(1)). When $S+T-R-P\neq0$, for a given $p$, there exists a unique $x^{*}(p)$ with

$x^{*}(p)=\frac{1}{2}+\frac{1-\omega(p^{2}+(1-p)^{2})}{S+T-R-P}\left[\frac{R-P}{2(1-\omega)}-\frac{T-S}{2(1-\omega(1-2p))}\right]$ (S19)

such that $dx/dt=0$ (note that $x^{*}(p)$ may not be in the interval $[0,1]$). It can be shown that $dx^{*}(p)/dp>0$ if $S+T-R-P>0$ and $dx^{*}(p)/dp<0$ if $S+T-R-P<0$. Thus, for a given $x$, there also exists a unique $p=p^{*}(x)$ (which is the inverse function of $x^{*}(p)$) such that $dx/dt=0$. In contrast, when $S+T-R-P=0$, Eq.(S17) is independent of $x$, and there exists a unique $p^{*}=(1-\omega)(P-S)/\omega(R-P)$ such that $dx/dt=0$. When $p\to0$, it is easy to check that $dx/dt<0$ for all $x\in(0,1)$ (i.e., $p^{*}(x)>0$ for all $x\in(0,1)$). On the other hand, when $p\to1$, it can be shown that $dx/dt>0$ for $x\to1$ (i.e., $p^{*}(1)<1$) if and only if $\bar{n}>(R-S)/(2R-S-T)$ (this condition holds if a TFT population cannot be invaded by any non-cooperative strategy). Thus, if $dx/dt>0$ for $p\to1$, $p=p^{*}(x)$ is a curve separating the $(x,p)$-plane such that $dx/dt>0$ for $p>p^{*}(x)$ and $dx/dt<0$ for $p<p^{*}(x)$.

We then look at the dynamic behavior of Eq. (S18) (i.e., the second equation of Eq.(1)). It is easy to see that for any $x\in(0,1)$, (i) if $R+P>S+T$, then $dp/dt<0$ for $p>1/2$ and $dp/dt>0$ for $p<1/2$ (i.e., $p$ always converges to $1/2$), (ii) if $R+P=S+T$, then $dp/dt=0$ and $p$ remains constant, (iii) if $R+P<S+T$, then $dp/dt>0$ for $p>1/2$ and $dp/dt<0$ for $p<1/2$ (i.e., $p$ converges to either $0$ or $1$).

The above analysis implies that the dynamics Eqs.(1)-(2) always have a continuum of (neutral) stable non-cooperative equilibria, $\{(0,p)|0\leq p<p^{*}(0)\}$, and a continuum of (neutral) stable cooperative equilibria, $\{(1,p)|p^{*}(1)<p\leq1\}$, exists if $\bar{n}$ is large enough such that a TFT population cannot be invaded by any non-cooperative strategy. Besides, the dynamics have two unstable equilibria, $(0,p^{*}(0))$ and $(1,p^{*}(1))$. Furthermore, a trajectory of the adaptive dynamics starting from $(1,p)$ with $p<p^{*}(1)$ will converge to a stable defective equilibrium if $R+P\leq S+T$. However, this trajectory is attracted by a stable cooperative equilibrium if $R+P>S+T$ and $p$ is only slightly smaller than $p^{*}(1)$.

**B. The repeated Public Goods Game**

**Payoff calculation**

We consider a repeated $m$-person PGG, where one player (i.e., the mutant, called player 1) uses $S_{1}=(x_{1},p_{1})$ and the other $m-1$ players (i.e., the residents, called player 2) use $S_{2}=(x_{2},p_{2})$. Again, we assume that after each round there is a probability $\omega$ ($0\leq\omega<1$) that another round will be played, i.e., the expected number of rounds is $\bar{n}=1/(1-\omega)$. Denote the contribution rate of player $i$ in round $t$ by $y_{i}(t)$ ($i=1,2$). Thus, in the first round, we have $y_{1}(1)=x_{1}$ and $y_{2}(1)=x_{2}$. In round $k+1$, $y_{1}(k+1)$ and $y_{2}(k+1)$ satisfy the following equations

$\begin{matrix} y_{1}(k+1)=y_{1}(k)+\left(y_{2}(k)-y_{1}(k)\right)p_{1}=\left(1-p_{1}\right)y_{1}(k)+p_{1}y_{2}(k)\text{，} \\ y_{2}(k+1)=y_{2}(k)+\left(\frac{\left(m-2\right)y_{2}(k)+y_{1}(k)}{m-1}-y_{2}(k)\right)p_{2}=\frac{m-1-p_{2}}{m-1}y_{2}(k)+\frac{p_{2}}{m-1}y_{1}(k). \end{matrix}$ (S20)

We next derive the expressions of $y_{1}(k)$ and $y_{2}(k)$. Suppose that

$y_{1}(k+1)+\alpha y_{2}(k+1)=\beta(y_{1}(k)+\alpha y_{2}(k)),$ (S21)

where $\alpha$ and $\beta$ are undetermined coefficients. From Eq.(S20), we obtain $\alpha=(m-1)p_{2}/p_{1}$ and $\beta=1$. Thus, $y_{1}(k)$ and $y_{2}(k)$ satisfy the following recursive equations

$\begin{matrix} y_{1}(k+1)=\left(1-p_{1}-\frac{p_{2}}{m-1}\right)y_{1}(k)+x_{2}p_{1}+\frac{x_{1}p_{2}}{m-1}\text{，} \\ y_{2}(k+1)=\left(1-p_{1}-\frac{p_{2}}{m-1}\right)y_{2}(k)+x_{2}p_{1}+\frac{x_{1}p_{2}}{m-1}. \end{matrix}$ (S22)

and the general formulas of $y_{1}(k)$ and $y_{2}(k)$ can be obtained

$\begin{matrix} y_{1}(k)=\frac{(m-1)x_{2}p_{1}+x_{1}p_{2}}{(m-1)p_{1}+p_{2}}+\left(1-p_{1}-\frac{p_{2}}{m-1}\right)^{k-1}\frac{(m-1)\left(x_{1}-x_{2}\right)p_{1}}{(m-1)p_{1}+p_{2}}, \\ y_{2}(k)=\frac{(m-1)x_{2}p_{1}+x_{1}p_{2}}{(m-1)p_{1}+p_{2}}-\left(1-p_{1}-\frac{p_{2}}{m-1}\right)^{k-1}\frac{(x_{1}-x_{2})p_{2}}{(m-1)p_{1}+p_{2}}. \end{matrix}$ (S23)

Let us now calculate the expected payoffs for the two types of players. From Eq.(S23), the total contributions of player 1 and player 2 in the repeated PGG are $\sum_{n=1}^{\infty} (1-\omega)\omega^{n-1}\sum_{k=1}^{n} y_{1}(k)$ and $\sum_{n=1}^{\infty} (1-\omega)\omega^{n-1}\sum_{k=1}^{n} y_{2}(k)$, respectively. Thus, $E(S_{1})$ and $E(S_{2})$ are written as

$E(S_{1})=\frac{\left(r-1\right)\left((m-1)x_{2}p_{1}+x_{1}p_{2}\right)}{\left(1-\omega\right)\left((m-1)p_{1}+p_{2}\right)}+\frac{\left(x_{1}-x_{2}\right)(m-1)\left((r-m)p_{1}-rp_{2}\right)}{m\left((m-1)p_{1}+p_{2}\right)\left(1-\omega\left(1-p_{1}-\frac{p_{2}}{m-1}\right)\right)},$ (S24)

and

$E(S_{2})=\frac{\left(r-1\right)\left((m-1)x_{2}p_{1}+x_{1}p_{2}\right)}{\left(1-\omega\right)\left((m-1)p_{1}+p_{2}\right)}+\frac{\left(x_{1}-x_{2}\right)(m-1)\left(r(p_{1}-p_{2})-\frac{mp_{2}}{m-1}\right)}{m\left((m-1)p_{1}+p_{2}\right)\left(1-\omega\left(1-p_{1}-\frac{p_{2}}{m-1}\right)\right)},$ (S25)

respectively. Furthermore, when $x_{1}=x_{2}=x$, $E(S_{1})=E(S_{2})=\bar{n}(r-1)x$.

**Evolutionary stability of TFT**

Consider a large population that consists of TFT resident (i.e., $S_{2}=(1,1)$). The average payoff of this population is$\bar{n}(r-1)$. This population can prevent the invasion of a non-cooperative strategy $S_{1}=(x_{1},p_{1})$ with $x_{1}<1$ if $E(S_{1})<\bar{n}(r-1)$. From Eq.(S24), $E(S_{1})<\bar{n}(r-1)$ is equivalent to

$p_{1}\left[(m-1)(m-r)(1-\omega)-(r-1)m\omega\right]-(r-1)m\left(1-\omega\frac{m-2}{m-1}\right)-r(m-1)(1-\omega)>0,$ (S26)

where this condition is independent of $x_{1}$. Notice that the left hand side of inequality Eq.(26) is a linear function of $p_{1}$, $E(S_{1})<\bar{n}(r-1)$ for all $p_{1}\in[0,1]$ and $x_{1}\in[0,1)$ if and only if the TFT population can prevent the invasion of the two extreme strategies AllD $(0,0)$ and STFT $(0,1)$. For both $S_{1}=(0,0)$ and $S_{1}=(0,1)$, it is easy to show that $E(S_{1})<\bar{n}(r-1)$ holds if and only if $\bar{n}>(m^{2}-2m+r)/(r-1)m$. In particular, when $m=2$, this condition (i.e., $\bar{n}>r/2(r-1)$) is equivalent to that a TFT population cannot be invaded by any non-cooperative strategy in a discrete PD game with payoff values $R=r-1$, $S=r/2-1$, $T=r/2$ and $P=0$.

**Evolutionary dynamics**

Similarly as Section A in S1 Text, we consider a large homogeneous population with (resident) strategy $(x,p)$, and assume that the population moves towards the direction where mutants have the higher invasion payoff. Then the resulting adaptive dynamics is given by

$\begin{matrix} \frac{dx}{dt}=\frac{\partial E(S_{1})}{\partial x_{1}}\left|_{x_{1}=x_{2}=x,p_{1}=p_{2}=p}\right.=\frac{\left(r-1\right)}{\left(1-\omega\right)m}-\frac{(m-1)}{m\left(1-\omega\left(1-\frac{pm}{m-1}\right)\right)}, \\ \frac{dp}{dt}=\frac{\partial E(S_{1})}{\partial p_{1}}\left|_{x_{1}=x_{2}=x,p_{1}=p_{2}=p}\right.=0, \end{matrix}$ (S27)

with two boundary conditions (i) $dx/dt\left|_{x=0}\right.=0$ if $dx/dt\left|_{x=0}\right.<0$ and (ii) $dx/dt\left|_{x=1}\right.=0$ if $dx/dt\left|_{x=1}\right.>0$. From Eq.(S27), $p$ remains constant and there exists a unique $p^{*}=(1-\omega)(m-1)(m-r)/\omega m(r-1)$ such that $dx/dt=0$. Similarly as the dynamic behavior of Eq.(2), there always exists a continuum of (neutral) stable defective equilibria, denoted by $\{(0,p)|0\leq p<p^{*}\}$, and a continuum of (neutral) stable cooperative equilibria, denoted by $\{(1,p)|p^{*}<p\leq1\}$, exists if a TFT population cannot be invaded by any non-cooperative strategy. Besides, $(0,p^{*})$ and $(1,p^{*})$ are two unstable equilibria. Furthermore, a trajectory of Eq.(3) (or Eq.(S27)) starting from $(x,p)$ converges to $(1,p)$ if $p>p^{*}$, and converges to $(0,p)$ if $p<p^{*}$.
